# Prevalence of inflicted and neglectful femur shaft fractures in young children in national level I trauma centers

**DOI:** 10.1007/s00247-022-05378-8

**Published:** 2022-05-07

**Authors:** Marie-Louise H. J. Loos, Roel Bakx, J. H. Allema, Frank W. Bloemers, Jan A. Ten Bosch, Michael J. R. Edwards, Jan B. F. Hulscher, Claudia M. G. Keyzer-Dekker, Egbert Krug, Victor A. de Ridder, W. Richard Spanjersberg, Arianne H. Teeuw, Hilco P. Theeuwes, Selena de Vries, Ralph de Wit, Rick R. van Rijn, The FurAHA study group, Anne de Boer, Esther Edelenbos, J. Carel Goslings, Lia P. G. W. van Sommeren, Annelies Toor, Jasmijn J. M. Verburg, Marjo Affourtit, Jan C. van Ditshuizen, Rene M. H. Wijnen, Dagmar R. J. Kempink, Johannes H. J. M. Bessems, Tjebbe Hagenaars, Dennis den Hartog, M. A. C. Jansen, A. P. A. Greeven, Floris E. P. Kanters, Annemieke Aalbers-Hiemstra, Arnaud Mulder, Frans Smiers, Rina C. Hartendorf, Audrey A. A. Fiddelers, Birgit Levelink, Martijn Poeze, Ivo de Blaauw, Tjarda N. Tromp, Benn Beuker, Inge Reininga, Klaus Wendt, Stasja J. G. Aspers-Wolters, Elise M. van de Putte

**Affiliations:** 1 Department of Paediatric Surgery, Emma Children’s Hospital, Paediatric Surgical Centre Amsterdam, Amsterdam UMC, University of Amsterdam & Vrije Universiteit Amsterdam, Meibergdreef 9, 1105 AZ Amsterdam, The Netherlands; 2 Department of Surgery, Haga Teaching Hospital & Juliana Children’s Hospital, The Hague, The Netherlands; 3 Department of Trauma Surgery, Amsterdam UMC, Vrije Universiteit Amsterdam, Amsterdam, The Netherlands; 4 Department of Trauma Surgery, Maastricht University Medical Centre, Maastricht, The Netherlands; 5 Department of Trauma Surgery, Radboud University Medical Centre, Nijmegen, The Netherlands; 6 Department of Surgery, Division of Paediatric Surgery, University Medical Centre Groningen, Groningen, The Netherlands; 7 Erasmus Medical Centre, Department of Paediatric Surgery, Sophia Children’s Hospital, Rotterdam, The Netherlands; 8 Department of Trauma Surgery, Leiden University Medical Centre, Leiden, The Netherlands; 9 Department of Paediatric Surgery, University Medical Centre Utrecht, Utrecht, The Netherlands; 10 Department of Trauma Surgery, Isala Clinics, Zwolle, The Netherlands; 11 Department of Social Paediatrics, Emma Children’s Hospital, Amsterdam UMC, University of Amsterdam, Amsterdam, The Netherlands; 12 Department of Trauma Surgery, Elizabeth TweeSteden Hospital, Tilburg, The Netherlands; 13 Department of Forensic Medicine, Section of Forensic Paediatrics, Netherlands Forensic Institute, The Hague, The Netherlands; 14 Department of Trauma Surgery, Medisch Spectrum Twente, Enschede, The Netherlands; 15 Department of Radiology and Nuclear Medicine, Emma Children’s Hospital, Amsterdam UMC, University of Amsterdam, Amsterdam, The Netherlands; 16 Amsterdam Center for Forensic Science and Medicine, Amsterdam, The Netherlands

**Keywords:** Child abuse, Children, Femur, Fractures, Infants, Inflicted trauma, Radiography

## Abstract

**Background:**

The prevalence of inflicted femur fractures in young children varies (1.5–35.2%), but these data are based on small retrospective studies with high heterogeneity. Age and mobility of the child seem to be indicators of inflicted trauma.

**Objective:**

This study describes other factors associated with inflicted and neglectful trauma that can be used to distinguish inflicted and neglectful from accidental femur fractures.

**Materials and methods:**

This retrospective study included children (0–6 years) who presented with an isolated femur fracture at 1 of the 11 level I trauma centers in the Netherlands between January 2010 and January 2016. Outcomes were classified based on the conclusions of the Child Abuse and Neglect teams or the court. Cases in which conclusions were unavailable and there was no clear accidental cause were reviewed by an expert panel.

**Results:**

The study included 328 children; 295 (89.9%) cases were classified as accidental trauma. Inflicted trauma was found in 14 (4.3%), while 19 (5.8%) were cases of neglect. Indicators of inflicted trauma were age 0–5 months (29%, positive likelihood ratio [LR +] 8.35), 6–12 months (18%, LR + 5.98) and 18–23 months (14%, LR + 3.74). Indicators of neglect were age 6–11 months (18%, LR + 4.41) and age 18–23 months (8%, LR + 1.65). There was no difference in fracture morphology among groups.

**Conclusion:**

It is unlikely that an isolated femur fracture in ambulatory children age > 24 months is caused by inflicted trauma/neglect. Caution is advised in children younger than 24 months because that age is the main factor associated with inflicted trauma/neglect and inflicted femur fractures.

**Supplementary Information:**

The online version contains supplementary material available at 10.1007/s00247-022-05378-8.

## Introduction

Consider a 14-month-old child admitted to the emergency department with a painful lower extremity. As physician, you question the caregiver, examine the child and order the diagnostic tests. The radiograph reveals a diaphyseal femur fracture and you question whether this fracture was caused by inflicted trauma (including neglect) or accidental trauma. In daily practice, the cause of fracture might not be clear and physicians have to decide whether further investigations are mandated (to help differentiate between accidental and inflicted trauma). To estimate the risk of inflicted trauma, one must have knowledge of relevant clinical features and the prevalence of inflicted trauma. Physicians must know which indicators increase or decrease the likelihood of inflicted trauma. The prevalence of inflicted/neglectful femur fractures in children varies (1.5–35.2%), with the highest in infants (< 12 months old) [1]. Yet, the prevalence is based on the results of a systematic review consisting of small retrospective studies with a high heterogeneity [1]. In our experience, children with a femur fracture are commonly referred for evaluation by the hospital-based Child Abuse and Neglect team. Although a femur fracture can be caused by a low-velocity accident, most physicians refer for suspected inflicted trauma evaluation irrespective of the ambulatory status of the child. Mitchell et al. [2] recommended a trauma evaluation for children younger than 18 months, while Son-Hing et al. [3] showed that there is little justification for a trauma evaluation in children older than 12 months. Son-Hing et al.’s statement was based on the fact that the incidence of inflicted trauma/neglect in children ages 13–36 months was similar to that in children ages ≥ 36 months [3]. According to a retrospective single-center study [4], the odds ratio (OR) of a femur fracture caused by inflicted trauma is 1.8 in infants (< 18 months), in contrast to 0.3 in toddlers (18 months to 4 years). In addition, it is important to note that the authors did not distinguish between pre-ambulatory and ambulatory infants in this study [4]. Age (ambulatory status) of the child seems to be an indicator of inflicted trauma, but it is not clear what other factors associated with inflicted trauma/neglect can be used to distinguish inflicted/neglectful from accidental femur fractures.

The aim of this study was to analyze the prevalence and factors associated with inflicted trauma/neglect among young children with an isolated femur fracture. We initiated a nationwide collaboration among all level I trauma centers in the Netherlands.

## Materials and methods

This was a retrospective study of children (ages 0–6 years) who presented with an isolated femur fracture at 1 of the 11 level I trauma centers in the Netherlands between January 2010 and January 2016. All hospitals were designated level I trauma centers [5]. The medical ethics review committee of the Academic Medical Center, Amsterdam, reviewed this study (reference number W17_121 #17.140, dated March 23, 2017) and concluded that no informed consent was required in accordance with national and international legislation. Boards and medical ethics review committees of participating hospitals reviewed and approved the study protocol. Data processing and storage were in accordance with European Union General Data Protection Regulation. Data were collected using an electronic data capture system (Castor EDC, 2019, Amsterdam, the Netherlands) [6].

### Definitions and outcomes

The primary outcome of this study was the prevalence of inflicted trauma/neglect among children (0–6 years) who presented with an isolated femur fracture at level I trauma center in the Netherlands. Secondary outcomes included the factors associated with inflicted trauma/neglect and clinical features of inflicted trauma/neglect compared with accidental trauma, including demographics and clinical history.

Definitions of inflicted trauma/neglect, accidental trauma and indeterminate are described in Online Supplementary Material 1. The definition of inflicted trauma/neglect is based on Dutch law and consistent with the definition of maltreatment formulated by the World Health Organization [7, 8].

The Child Abuse and Neglect team is a multidisciplinary team specializing in the evaluation of suspected cases of child abuse. The team includes a pediatrician specializing in social pediatrics, a pediatric radiologist, a pediatric surgeon, an emergency department physician/nurse and a physician from the national Child Protective Services. Some hospitals maintained permanent Child Abuse and Neglect teams that included a pediatrician specializing in social pediatrics, an emergency department physician/nurse and social workers. In these Child Abuse and Neglect teams, a trauma/pediatric surgeon, a radiologist and Child Protective Services were consulted if necessary.

### Data extraction

We collected clinical data, psychosocial information and findings from the Child Abuse and Neglect teams using a standardized case report form maintained in an electronic database [6]. We identified eligible patients, demographics and length of hospital stay from the Dutch National Trauma Registry [9]. 

Clinical information included type of femur fracture according to the AO classification [10] and Salter–Harris guideline [11], other injuries, detailed history and injury mechanism, and findings of the child abuse screening within the emergency department [12]. For the purposes of this study, all radiographs were reviewed and fractures classified by three researchers (R.R.vR., a pediatric radiologist with 19 years of experience; R.B., a pediatric surgeon with 12 years of experience; M-L.H.J.L., a medical doctor with 4 years of experience). Psychosocial information included details concerning family composition and socioeconomic status based on postal code data provided by Statistics Netherlands [13]. Child Abuse and Neglect team information included conclusions as to whether inflicted trauma/neglect was suspected/confirmed and whether further actions were required (e.g., social services consultation, foster care, report to police).

### Classification of cases

We included cases from the Dutch National Trauma Registry of children [14] ages 0–6 years who presented at one of the Dutch level I trauma centers with an isolated femur fracture as chief complaint during admission to the emergency department. Indeterminate cases were excluded from analysis.

To classify cases, we used conclusions from the Child Abuse and Neglect teams, the court and the expert panel. Cases were classified into one of three groups: accidental trauma, inflicted trauma/neglect or indeterminate. The definitions of accidental trauma, inflicted trauma/neglect and indeterminate are provided in Online Supplementary Material 1. All cases with a clear accidental cause (those observed by a third party and not associated with concerns regarding inflicted trauma) were classified as accidental trauma. The cases evaluated by Child Abuse and Neglect teams were assessed further. When Child Abuse and Neglect teams concluded that a child was injured by inflicted trauma/neglect, the child was referred to Child Protective Services. Some cases were referred to Child Protective Services without input from the Child Abuse and Neglect teams; feedback from Child Protective Services was used to classify these cases. The third group included cases in which the cause of injury was not evident and no Child Abuse and Neglect team/Child Protective Services input was available. These cases were evaluated by an expert panel that consisted of five members of the research group: a trauma surgeon (E.K., with 18 years’ experience), a pediatric surgeon (R.B., with 12 years’ experience), a forensic pediatric radiologist (R.R.vR., with 19 years’ experience), a forensic physician (S.dV., with 10 years’ experience) and a pediatrician specializing in social pediatrics (A.H.T., with 29 years’ experience). The expert panel discussed each of these cases and determined whether the injuries were accidental versus potentially inflicted or caused by neglect. Because the expert panel was reviewing these cases retrospectively, it was not always possible to draw definite conclusions regarding inflicted trauma based on the available information. Some cases lacked relevant additional information. These cases were classified as indeterminate.

### Statistical analysis

Data were analyzed using SPSS version 27 (IBM Corp., Armonk, NY). Quantitative variables were summarized as medians and interquartile ranges (IQR), while categorical variables were presented as counts and percentages. Three groups were used for the analysis: inflicted trauma, neglect and accidental trauma. The chi-square/Fisher exact test or Kruskal–Wallis test was used to identify statistically significant differences. Likelihood ratios (LRs) were calculated. A *P*-value less than 0.05 was considered statistically significant; 95% confidence intervals (CIs) were calculated as appropriate.

## Results

**Fig. 1:**
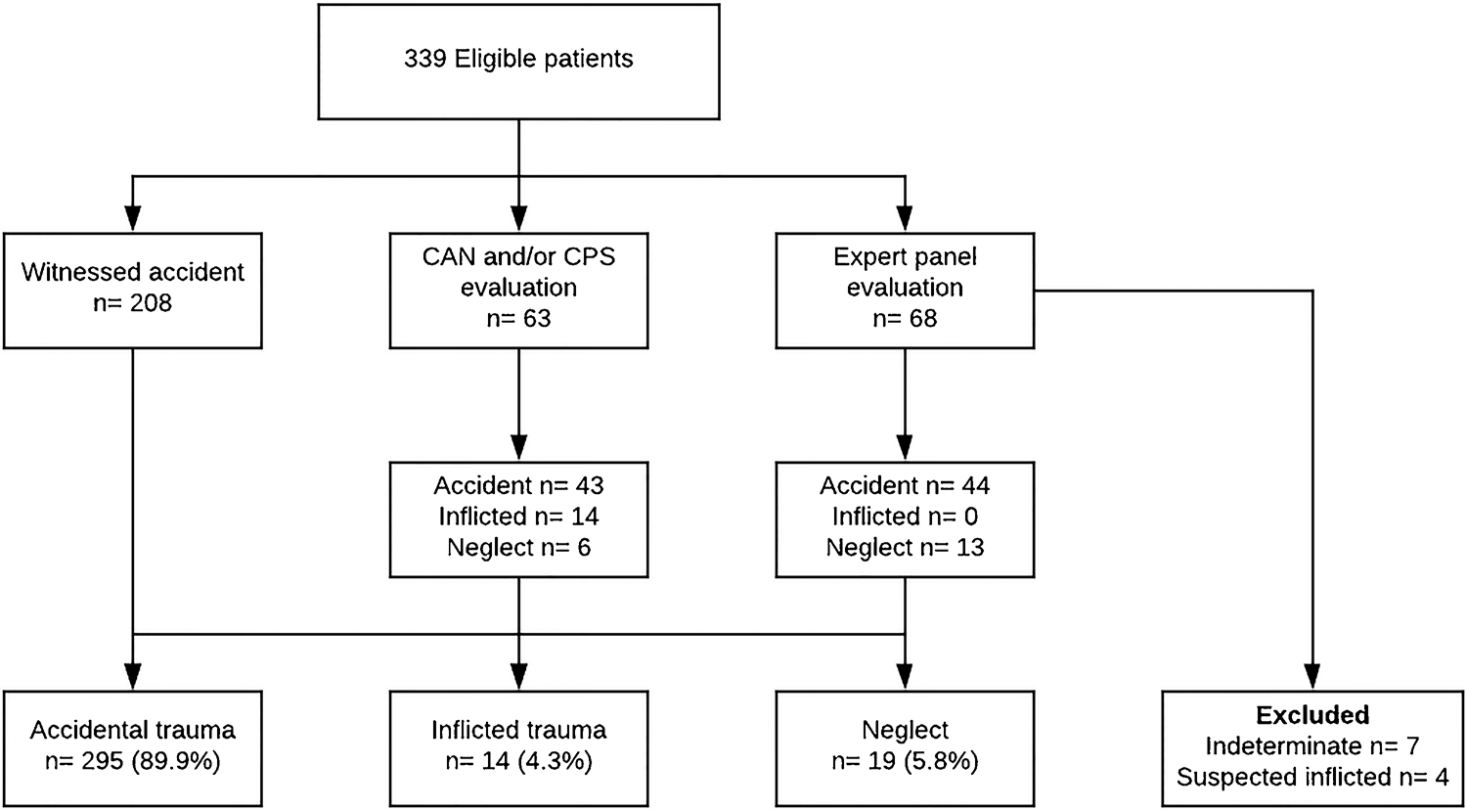
Classification of cases. *CAN* Child Abuse and Neglect team, *CPS* Child Protective Services

**Fig. 2:**
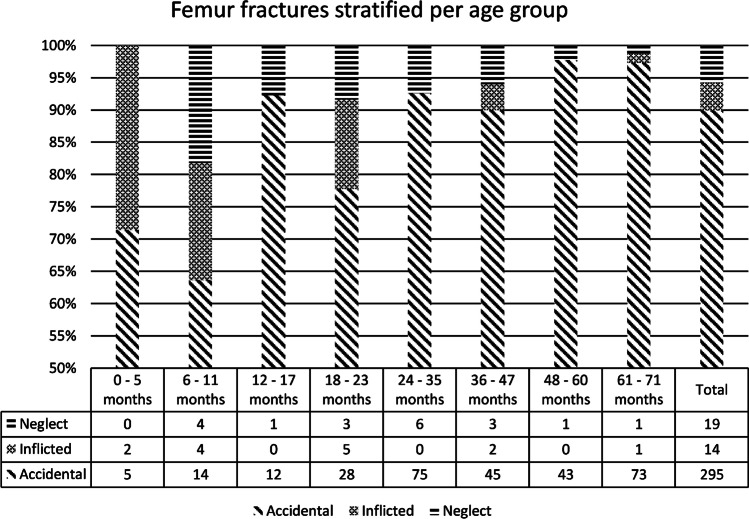
The number of children with a femur fracture stratified per age group and category. The y-axis starts at 50% to create a better overview

A total of 328 children with an isolated femur fracture were admitted to a level I trauma center. Fourteen (4.3%) were classified as inflicted trauma, 19 (5.8%) as neglect and 295 (89.9%) as accidental trauma (Fig. 1). An overview of demographic data and clinical parameters is provided in Table 1. There were 231 (70%) boys (median age 38 months, IQR 26–59 months) and 97 (30%) girls (median age 33 months, IQR 20–57 months). All children sustained an acute fracture. An overview of the children stratified by age group is provided in Fig. 2. Significantly more boys were included, but we found no gender distribution differences between groups.

**Table 1:** Demographic data and clinical parameters

	Accidental	Inflicted	Neglect	
*n* (%)	295 (89.9%)	14 (4.3%)	19 (5.8%)	*P*-value^a^
Age (months)				
Median (IQR)	39 (26–59)	19 (8–28)	29 (17–38)	** < 0.001**
Gender				
Male, *n* (%)	209 (71%)	12 (86%)	10 (53%)	0.11
Female, *n* (%)	86 (29%)	2 (14%)	9 (47%)	0.11
Socioeconomic status				
Low, *n*	88	5	7	0.76
Normal, *n*	138	9	7	0.29
High, *n*	66	-	5	0.12
Unknown, *n*	3	-	-	-
Trauma mechanism				
Fall while walking/running	89	4	3	0.41
Low fall (height < 1 m)	72	3	3	0.68
High fall (height > 1 m)^b^	55	4	9	**0.009**
Motor vehicle vs. pedestrian/cyclist	24	-	2	0.50
Trampoline	24	-	2	0.50
Entrapment	14	2	-	0.15
Motor vehicle crash	5	-	-	0.75
No trauma	3	1	-	0.11
Pull^c^	3	-	-	0.84
Other^d^	6	-	-	-
Hospitalization (days)				
Median (IQR)	5 (2–13)	15 (5–22)	8 (2–15)	0.13
ICU admission, *n*	5	1	-	0.94
Time of admission, *n*				
8 am–12 pm	51	2	3	0.95
12 pm–5 pm	113	5	11	0.23
5 pm–11 pm	103	3	5	0.45
11 pm–8 am	7	3	-	** < 0.001**
Unknown	21	1	-	-

*ICU* intensive care unit, *IQR* interquartile range

^a^ *P* < 0.05 is significant (bold)

^b^ High fall includes all children who fell downstairs from the arms of their caregiver (n = 10)

^c^ Pull includes situations where there was a pulling motion on the leg by someone other than the child (for example while playing or diaper change)

^d^ Unknown (*n* = 3), cycle versus cycle (*n* = 1), cycle versus pedestrian (*n* = 1) and ski accident (*n* = 1)

Of all included cases, a skeletal survey and head CT were performed in 20 cases and showed additional injuries in 2 children. Ophthalmology was conducted in 4 cases, and no retinal hemorrhage was detected.

An overview of the likelihood ratios (LR) of inflicted femur fractures stratified by age group is provided in Table 2. Inflicted trauma was more prevalent in infants (< 6 months old) (*n* = 2 [29%] inflicted versus *n* = 0 [0%] neglect versus *n* = 5 [71%] accidental; *P* = 0.005) with LR + 8.35. Inflicted and negligent trauma were more prevalent in children ages 6–11 months (*n* = 4 [18%] inflicted versus *n* = 4 [18%] neglect versus *n* = 14 [64%] accidental; *P* < 0.001) with LR + 5.98 (inflicted) and 4.41 (neglect) and in children ages 18–23 months (*n* = 5 [14%] inflicted versus *n* = 3 [8%] neglect versus *n* = 28 [78%] accidental, *P* = 0.008), with LR + 3.74 (inflicted) and 1.65 (neglect).

**Table 2:** Likelihood ratios of inflicted/neglectful femur fractures stratified by age group

Age (months)	Inflicted injury^a^		Neglect^a^	
	LR +	LR -	LR +	LR -
0–5	8.35	0.87	-	-
6–11	5.98	0.75	4.41	0.83
12–17	-	-	1.28	0.99
18–23	3.74	0.71	1.65	0.93
24–35	-	-	1.23	0.88
36–47	0.93	1.01	1.03	0.99
48–60	-	-	0.36	1.11
61–71	0.29	1.23	0.22	1.25

^a^*LR* + is the positive likelihood ratio and is based on the formula: sensitivity / 1–specificity; *LR–* is the negative likelihood ratio and is based on the formula: 1–sensitivity / specificity

### Factors associated with inflicted trauma/neglect

The most commonly occurring injury mechanism was fall while walking or running, which caused an isolated femur fracture in 30% of accidental trauma cases. A high fall (from height > 1 m) was most frequently caused by neglect (29% inflicted, 47% neglect, 19% accidental trauma, *P* = 0.009); other trauma mechanisms did not differ among the groups (Table 1).

The proportion of children with an inflicted femur fracture admitted to the emergency department during night hours (11 pm–8 am) was greater than the proportion of those with accidental femur fracture; no differences were found in duration of hospitalization (Table 1).

Fourteen children sustained an inflicted fracture. Details of these 14 children are provided in Online Supplementary Material 2. Nineteen children sustained a femur fracture because of neglect, and these are detailed in Online Supplementary Material 3. None of the children in the inflicted/neglect groups were reported as having a physical disability, in contrast to 10 children in the accidental group.

### Fracture morphology

An overview of fracture morphology is provided in Table 3. Most fractures were mid-diaphyseal (88%). The most common type was a spiral fracture in 168 cases (51%; AO 32.A1), followed by transverse fracture in 64 (20%; AO 32.A3) and oblique fracture in 49 (15%; AO 32.A2). There were no significant differences in fracture morphology among the groups.

**Table 3:** Morphology of femur fractures stratified by category

*n*	Accidental	Inflicted	Neglect	*P*-value^a^
	295	14	19	
Proximal fracture	10	1	1	0.61
Mid diaphyseal	262	10	16	0.15
AO 32.A1 (spiral)	153	7	8	0.65
AO 32.A2 (oblique)	44	2	3	0.75
AO 32.A3 (transverse)	59	1	4	0.52
Other^b^	6	-	1	-
Distal fracture	16	2	2	0.26
Not classified^c^	7	1	-	-

^a^ *P* < 0.05 is significant

^b^ Other types of fractures included wedge fractures of femur shaft AO 32.B1 (*n* = 1), AO 32.B2 (*n* = 4), AO 32.B3 (*n* = 2)

^c^ The morphology of eight femur fractures was unclear because there were missing radiographs caused by transition to electronic patient files; therefore the researchers were unable to assess the radiographs (only the radiologic report was available for these eight)

## Discussion

This study showed that the prevalence of inflicted trauma /neglect in children ages 0–6 years with an isolated femur fracture was 10.1% — 4.3% inflicted trauma and 5.8% neglect. The prevalence of inflicted trauma/neglect was 34.5% in children younger than 12 months (20.7% inflicted trauma) and 24.3% in children younger than 24 months (14.1% inflicted trauma) in contrast to children older than 24 months (5.6% inflicted trauma; 1.2% neglect). The main factor associated with inflicted trauma/neglect was the age of the child, which is in accordance with two previously published systematic reviews [1, 15]. The proportion of inflicted trauma was highest in children younger than 12 months [1], but neglect was not included. Other studies recommend a trauma evaluation of children younger than 18 months (if the trauma was not independently verified) because the prevalence of inflicted trauma/neglect is high (25–34%) [4, 16, 17]. Our study also showed a peak of inflicted trauma/neglect at 18–23 months old, which is not supported by other studies and perhaps less recognized because these children are usually able to walk. Although Leventhal et al. [18] found similar results in their study regarding fractures and inflicted trauma/neglect, children were more likely to sustain a fracture caused by inflicted trauma/neglect if younger than 12 months (39%) and less likely if older than 23 months (8%).

Physicians associate femur fractures in children with inflicted trauma and commonly refer them for evaluation by Child Abuse and Neglect teams. Although not recognizing inflicted trauma/neglect has serious consequences, an incorrect referral can have vexing consequences for the child and caregivers. We try to put this into perspective. To automatically consider inflicted trauma in the event of a femur fracture in children is not entirely justified — young children can sustain a femur fracture from low-velocity trauma. Schwend et al. [19] showed that inflicted femur fractures were found in 42% of non-ambulatory children compared to 2.6% in ambulatory children and concluded that it is very unlikely that a femur fracture is caused by inflicted trauma in ambulatory children.

The prevalence of inflicted femur fractures is comparable to that of a toddler’s fracture (tibia fracture). Coffey et al. [16] showed that in 55 children < 18 months old with a lower-extremity fracture, 22 had a unilateral and 6 had a bilateral *femur* fracture versus 14 unilateral and 9 bilateral *tibia* fractures caused by inflicted trauma. In the accidental trauma group, 13 had a femur fracture and 1 a tibia fracture. Worlock et al. [20] showed similar results: in infants, they found 5 femur and 5 tibia fractures in the inflicted trauma group, versus 2 femur and 1 tibia fracture in the accidental trauma group. Although there is limited evidence regarding toddler’s fractures in relation to inflicted trauma, it is generally accepted as an accidental injury [21–23].

Several children sustained a femur fracture following a fall from a height, e.g., fall off a jungle gym, fall down the stairs or off a dresser/table. Regarding the reported injury mechanisms, only fall from height was related to inflicted trauma/neglect, specifically neglect. Ghanem et al. [24] showed similar results; in their study, the main cause of lower-extremity fractures was neglect leading to a fall from a height. In our study, mechanisms were a fall off a dresser/table and a fall down the stairs. Regarding a fall off the dresser/table, this occurred when the caregiver was briefly distracted, and in combination with his/her (unexpected) developing motor skills, the child fell off a table in an unprotected moment. This phenomenon and call for attention has been described by Wegmann et al. [25], who found that most fractures in young children (< 12 months) were caused by a fall off a dresser, from the arms of caregivers or out of bed. Little is known regarding falls from the arms of caregivers and the occurrence of pediatric femur fractures in these instances [25].

Our data show that diaphyseal fractures were the most frequent location and spiral fractures (AO 32.A1) the most commonly occurring type. Our data show, in accordance with the systematic review of Wood et al. [1], that fracture morphology was not a factor associated with inflicted trauma/neglect. However, it is known that physicians have difficulty in differentiating fracture morphology [26] and are biased by fracture morphology. In another study, we investigated the influence of contextual information on the interpretation of radiographs of pediatric femur fractures, asking the physician to estimate the likelihood of inflicted trauma as the cause of the fracture [27]. Physicians indicated least suspicion of inflicted trauma if it concerned a transverse fracture. A diaphyseal fracture is associated with high-energy trauma (motor vehicle accidents) or fall from height [28]. Most femur fractures in our study were caused by a variety of low-velocity mechanisms. Capra et al. [29] concluded that femur fractures can be caused by low-velocity incidents in young healthy children. Ambrose et al. [30] investigated the biomechanical aspects of bones of 53 infants and used rib and tibia specimens. Samples were tested using a three-point bending method. Strength and stiffness increased with age (infant bones have 50% of the strength and 10–25% of stiffness compared to adults) and ductility decreased with age, with a peak at 4–5 months old (infant bones were 6 times more ductile than adult bones) [30]. As a consequence, infant bones do not need much force to be fractured and femur fractures in children can be caused by low-velocity incidents or by an indirect impact [31]. Spiral fractures are not specific for inflicted trauma/neglect; their presence merely implies that fracture was caused by a torsional force and may be a result of minimal force [1, 32]. Pierce et al. [33] used a biomechanical model on femur fractures in children who fell down the stairs and showed that transverse and short oblique fractures were associated with almost tenfold higher injury biomechanical measures compared to spiral and bucket handle fractures.

### Strengths

This study reports inflicted trauma/neglect in a large group of young children with an isolated femur fracture and is the first Dutch level I trauma center study, thanks to the cooperation of all level I trauma centers. Based on these data, we were able to identify the prevalence and factors associated with inflicted trauma/neglect. This information is useful for physicians and valuable for Child Abuse and Neglect teams regarding trauma evaluation. Another strength is that these results are generalizable for young children with an isolated femur fracture in other hospitals. In general, children with an isolated femur fracture are not referred to a level I trauma center for treatment; our included cases are similar to other young children with an isolated femur fracture in other hospitals. The advantage of conducting this research in level I trauma centers is that these hospitals have well-functioning Child Abuse and Neglect teams, which increases the reliability of the study results. Further, the Dutch National Trauma Registry data are of high quality and are prospectively collected by dedicated data managers.

### Limitations

As described by Taitz et al. [34] and Boyce et al. [35], we noticed that information in medical files was usually very brief and that essential information regarding the injury mechanism and inflicted trauma risk factors was often missing. Clues for inflicted trauma were missed because of the scarce documentation, “fall from dresser, femur fracture, cast immobilization” does not justify the patient’s treatment and trauma evaluation. Therefore, we had to classify some cases as indeterminate because we did not have data that would permit the expert panel to reach specific conclusions. Furthermore, we were unable to identify household characteristics that might be associated with inflicted trauma. We were able to provide a rough estimate of socioeconomic status based on the child’s residential neighborhood. In addition, we were only able to report sparse information regarding the ambulatory level of the child, namely only if the child was known to have a disability.

Another pitfall is the lack of performance of additional investigations. We noticed incomplete adherence to the contemporaneous guidelines recommending that additional investigations should be performed [36]. In addition, in four cases the expert panelists concluded that they would have initiated a full trauma evaluation if they had been consulted at the time. These cases had to be classified as indeterminate because of missing additional information. This might have led to an underestimation of inflicted trauma. Another limitation is the lack of a diagnostic gold standard. We attempted to use a diagnostic-type method using the information provided by Child Abuse and Neglect teams, Child Protective Services and the consensus opinions of the expert panel. While we considered the conclusions of the Child Abuse and Neglect team to be accurate, in most cases we did not have insight into the specific information used by the Child Abuse and Neglect team to generate its conclusions. This might have led to an overestimation of inflicted trauma/neglect. Moreover, both the Child Abuse and Neglect teams and the expert panel considered injuries in their assessments. This might have resulted in circular reasoning in some cases; however, availability of additional investigations was limited and injuries could have been missed. Nonetheless, consensus opinions of expert panels have been identified as a reliable means of assessing cases [37].

## Conclusion

In this nationwide level I trauma center study, we identified inflicted trauma/neglect in 10% of children with an isolated femur fracture during a 5-year period, of whom 4.3% were inflicted trauma and 5.8% neglect. An isolated femur fracture in ambulatory children was not specific for inflicted trauma/neglect, but caution is advised in children < 24 months old because of the high prevalence of inflicted trauma/neglect in this age group (24.3%). The results might be helpful in the evaluation of suspicious trauma by Child Abuse and Neglect teams. Finally, a substantial proportion of femur fractures was caused by a fall from a height, mostly by a fall from the arms of an adult or off a dresser or table. This trauma mechanism should be addressed in prevention campaigns to warn caregivers and to prevent femur fractures in young children.

## Supplementary Information

Below is the link to the electronic supplementary material.

Supplementary file1 (PDF 90 KB)

Supplementary file2 (PDF 66 KB)

Supplementary file3 (PDF 64 KB)
