# Supplementary material for: Prevalence of inflicted and neglectful femur shaft fractures in young children in national level I trauma centers
Source: Pediatr Radiol. 2022 May 7;52(12):2359–67. doi: 10.1007/s00247-022-05378-8 (PMC9616777; doi:10.1007/s00247-022-05378-8)
Supplement: Supplementary file 1 — Supplementary file1 (PDF 90 KB) [file 247_2022_5378_MOESM1_ESM.pdf]

Online Supplementary Material 1

Non-accidental trauma includes any form of physical interaction that is threatening or violent to a minor, actively or passively urged by the caregivers or other persons towards whom the minor is in a relationship of dependence or lack of freedom, that results in serious harm or the threat of serious harm to the minor in the form of physical injury. Non-accidental injuries are injuries as a result of non-accidental trauma.

Non-accidental trauma includes the following:

(1) *Inflicted trauma*: injuries resulting from the force of impact via the direct actions of someone other than the child him or herself, regardless of motive.

(2) *Neglect*: injuries resulting from the failure to protect a child from physical harm. Neglect was scored based on two domains, including supervision and environment. In other words, if either the environment or supervision were deemed to be inadequate, the case was classified as neglect (Below in this box a detailed description of these parameters).

The definition of neglect is based on national and international legislation [1,2]. The definition of neglect: The caregiver or person responsible for the child (parent) does not take suitable precautions to ensure the safety of the child indoors and/or outdoors according to the nature and development of the child. This includes allowing the child to be exposed to dangerous situations (e.g. allowing the child to play in unsafe places or leaving the child with a known violent person), as well as failing to familiarize themselves in advance regarding situations that are potentially dangerous to the child (e.g. no inquiry about the background or suitability of a nanny).

Two domains were ranked to determine the quantity of neglect per case, namely supervision and environment. If either the environment or supervision were inadequate, the case was classified as neglect. The age and level of development of the child were weighed for each domain, e.g. a very young (<1 year of age) child requires more intensive supervision than an older child. We agreed that around the age of 4 or 5 years, a scaled decrease of supervision was considered as age-appropriate, after which this decreases further in proportion according to the safety of the environment and the development of the child. The responsibility shifts gradually to the child until it becomes an adult.

1. (Lack of) supervision: Failure to take measures to ensure the safety of the child regarding supervision of the child. As the risk of accidents increase with the length of an unsupervised child, the greater this domain was ranked depending on the duration of the inadequate supervision. This includes lack of babysitting in the absence, illness or serious stress of the parents. Further, when parents cannot adequately monitor the safety of the child because they are under the influence of alcohol or drugs, or when caregivers have psychiatric problems that make them very unlikely to be able to supervise their children adequately.
2. (Un)safety of environment: Failure to ensure that the environment where the child is present or playing, is a safe place. It concerns an environment, both indoors and outdoors, that poses a direct physical danger to the child, such as the presence of cup of hot tea or candles near the child, broken glass or window, unsecured sockets, electrical wiring or toxic substances. The expert panel took into account that a described situation could be primarily unsafe or became unsafe due to an unforeseen circumstance.

In addition, risk factors concerning the household (such as single parent family) were not included in the assessment, as was the severity of the injury.

Accidental trauma: injuries in which there was no reasonable suspicion of inflicted trauma or neglect. The trauma mechanism was considered to be accidental by society at large and based on standard unwritten laws and definitions. In these cases, trauma was frequently observed by a person other than the primary caregivers (i.e., a "witnessed accident") and did not raise any concern for non-accidental trauma.

Indeterminate: injuries in which there was insufficient information available. In these cases, the cause of injuries sustained could not be determined and it was not possible to classify the case in one of the three aforementioned categories.

[1] World Health Organization. Child Maltreatment 2019, accessed 01-11-2021; [Online] available at: https://www.acf.hhs.gov/cb/report/child-maltreatment-2019 2019.

[2] article 1.1 Dutch Youthlaw (art. 1.1 Jeugdwet), accessed 01-12-2021, [Online] available at: https://wetten.overheid.nl/BWBR0034925/2018-01-01.
