# Supplementary material for: Prevalence of inflicted and neglectful femur shaft fractures in young children in national level I trauma centers
Source: Pediatr Radiol. 2022 May 7;52(12):2359–67. doi: 10.1007/s00247-022-05378-8 (PMC9616777; doi:10.1007/s00247-022-05378-8)
Supplement: Supplementary file 2 — Supplementary file2 (PDF 66 KB) [file 247_2022_5378_MOESM2_ESM.pdf]

Online Supplementary Material 2

A total of 14 children sustained an isolated femur fracture due to inflicted trauma. All 14 children were diagnosed with an isolated femur fracture, additional investigations revealed the following additional details of the cases:

1 + 2: Case #1 was an 11 months old boy, presented with a distal femur fracture (AO 33A2). The skeletal survey showed healing rib and a midshaft tibia fracture. Case #2 was an 1 month old boy (one of twins), presented with a midshaft femur fracture (spiral, AO 32.A1). There were healing humeral, skull and rib fractures detected on the skeletal survey, the ophthalmology did not reveal retinal haemorrhage. No other (somatic) causes for the fractures were found.

3 – 5: In 3 cases the caregiver admitted to have caused the fracture by using force on the child. Case #3 was a 5 year and 8 months old boy, sustaining a midshaft femur fracture (oblique AO 32.A2). According to his caregiver (female) the boy behaved annoyingly (dropped a glass of water). The caregiver kicked him because of this, after this, his caregiver bumped into him while he wanted to turn around and fell down. Case #4 was a boy aged 41 months old and sustained a midshaft femur fracture (spiral, AO 32.A1). According to his caregiver (female) he behaved badly (smashed a plate). His caregiver wanted to put him in time-out on the stairs, but did this too harshly, as a result of which he fractured his femur. Case #5 was a boy aged 47 months old and sustained a proximal femur fracture (transverse AO 31.A3). According to his caregiver (male), the boy was sitting on the stairs (about 1m height). The caregiver tried to grab the boy because of the boy refused to listen to the caregiver. By trying to grab him, the boy fell of the stairs and fractured his leg. A full head-to-toe physical exam of the boy revealed bruises located at his lower legs and one at his upper arm on the right.

6 – 10: The other cases did not have additional injuries, however the reported trauma was not compatible with the injury (n=5). Case #6 concerns a 7 month old boy sustaining a distal femur fracture (metaphyseal corner fracture, Salter Harris type 2) by a fall from the stairs out of the arms of the caregiver. Case #7 concerns a 19 month old girl sustaining a midshaft femur fracture (spiral, AO 32.A1) because she got entrapped between the floor and a heavy granite plate/wall that surrounds the bath and fell onto her (her caregiver could not lift it because of the heavy weight). Case #8 concerns a 18 month old boy sustaining a midshaft femur fracture (spiral, AO 32.A1) because his caregiver hold him in his arms, bumped into the door and put the boy on the stairs. Thereafter the boy fell of the stairs. Case #9 concerns a 21 month old boy sustaining a midshaft femur fracture (spiral, AO 32.A1) because he fell of a little bench (maximum 30cm height) at day-care. Case #10 concerns a 3 weeks old boy (one of twins) sustaining a midshaft femur fracture (oblique, AO 32.A2) because his caregiver (female) held him on her arm, lost her balance, pressed the boy against her breast after which she heard a snap. No other (somatic) causes for the fractures were found.

11 + 12: There were concerns about the interaction between caregiver and child. Case #11 concerns a 9 month old boy, who was put on a high closet (about 2m height) by his drunken caregiver (male). The boy fell of the closet and fractured his leg, midshaft femur fracture (transverse, AO 32.A3). #12: This boy was 23 months old, sustained a midshaft femur fracture (spiral, AO 32.A1), and presented also with bruises located at his back and face (cheek).

13 + 14: These cases were referred to Child Protective Services (n=2) in the past because of non-accidental trauma. Case 13 was an 8 month old girl, the type of fracture remains unclear due to the missing radiograph. Her caregiver (female) dropped her into the bath tub. The caregiver is mild mentally retarded. Case 14 concerns a 22 month old boy sustaining a midshaft femur fracture (spiral, AO 32.A1). The boy was in another room with is 5 year old brother when his parents suddenly heard crying. They do not know how the fracture was caused. The boy sustained two other unwitnessed fractures in the past (clavicle and tibia). This family was already supervised by Child Protective Services at time of this incident.

In cases 6 – 14 additional evaluation by Child Protective Services endorsed the diagnosis of inflicted trauma.
