# Supplementary material for: Prevalence of inflicted and neglectful femur shaft fractures in young children in national level I trauma centers
Source: Pediatr Radiol. 2022 May 7;52(12):2359–67. doi: 10.1007/s00247-022-05378-8 (PMC9616777; doi:10.1007/s00247-022-05378-8)
Supplement: Supplementary file 3 — Supplementary file3 (PDF 64 KB) [file 247_2022_5378_MOESM3_ESM.pdf]

Online Supplementary Material 3

Neglect was assessed by two domains: supervision and environment (Box 1).

Inappropriate supervision (n=11); case #1 – 11.

This concerns #1 a 6 year old girl sustaining a midshaft wedged femur fracture (AO 32.B3), because she fell with her bike on the streets while cycling to school. There were no caregivers present, she was brought back home by a stranger. #2 Concerns a 38 month old girl sustaining a midshaft femur fracture (transverse, AO 32.A3) because she fell of a balcony (3m height) while she was playing and climbed over the bars. Nobody witnessed the accident, she was found on the ground by neighbours. Case #3 concerns a 29 month old girl sustaining a midshaft femur fracture (transverse, AO 32.A3), because she fell out of an open window (4m height). Her caregiver left the window open after cleaning. Nobody witnessed the accident, she was found on the ground by neighbours. Case #4 concerns an 11 month old girl sustaining a midshaft femur fracture (transverse, AO 32.A3) because she fell down the stairs (from the first floor to ground floor). Nobody witnessed the accident, she was found at the bottom of the stairs by her caregiver who heard her crying. Case #5 concerns a 17 month old boy sustaining a midshaft femur fracture (spiral AO A32.A1) because he was put on a big trampoline by his caregivers, was launched of this by older children and fell next to the trampoline on the ground. No adults witnessed the accident, an older boy reported this incident to the parents. Case #6 concerns a 32 month old boy sustaining a midshaft femur fracture (spiral, AO 32.A1) because he fell into a well while playing. He managed to climb out himself, however his brother (<5 years old) had to carry him home, because there was no caregiver present. Case #7 concerns a 40 month old boy sustaining a proximal femur fracture (AO 31.B1) because he fell out of a window at the first floor (4m height). He was playing with is sister (<5 years old), she probably opened the window. Nobody witnessed the accident, he was found outside on the ground by his caregiver who heard him crying. Case #8 (an 8 month old boy, midshaft femur fracture, spiral, AO 32.A1), case #9 (10 month old girl, distal femur fracture, AO 33.A2), #10 (8 month old boy, distal femur fracture, AO 33.A2) and case #11 (38 month old girl, midshaft femur fracture, oblique OA 32.A2), all of them sustained a femur fracture because they fell of the dresser while the caregiver was (briefly) distracted and stepped away from the dresser or walked out of the room.

Dangerous environment (n=1); case 12.

This concerns a 25 month old girl sustaining a midshaft femur fracture (oblique OA 32.A2) because she slipped out of her car seat and fell down. Her caregiver put her in this car seat, although the caregiver knew it was inappropriate for this girl because it did not fit the girl.

Combination of inappropriate supervision and dangerous environment (n=5); case #13 – 17.

Case #13 concerns a 4 year old boy sustaining a transverse femur fracture (transverse AO 32.A3) because he was runover by a slow speeding backward driving car. He got out of the car and his caregiver (driver) lost him out of sight while he was parking the car. Case #14 concerns a 30 month old boy sustaining a midshaft femur fracture (oblique, AO 32.A2) because he was hit by a big truck while the patient was driving around on his toy truck at the property. There were no caregivers to watch him. Case #15 concerns an 23 month old boy sustaining a midshaft femur fracture (spiral, AO 32.A1) because he sneaked out with his sister and played on a trampoline at the neighbours. Nobody witnessed the accident, he was found next to the trampoline by the neighbour who heard him crying. Case #16 concerns a 32 month old boy sustaining a midshaft femur fracture (spiral, AO 32.A1) because he was entrapped between the ground and heavy wooden plates after the plates fell down on him. Nobody witnessed the accident, he was found below the plates by his caregiver who heard him crying. Case #17 concerns a 22 month old girl sustaining a midshaft femur fracture (spiral, AO 32.A1) because she fell down the stairs. Her caregiver left the stair gate open while he went downstairs to grab a diaper. She went after him and fell down the stairs.

In two cases there were other reasons to report the children to Child Protective Services. Case #18 concerns a 33 month old boy (midshaft spiral fracture, AO 32.A1) and case #19 a 23 month old girl (midshaft spiral fracture, AO 32.A1). These two children presented at the emergency department in a very unkempt condition, where signs of general physical neglect had already been observed. Most likely these cases fractured their femur due to an unsafe environment at home.
